# Spacer Cation Alloying in Ruddlesden–Popper Perovskites for Efficient Red Light‐Emitting Diodes with Precisely Tunable Wavelengths

**DOI:** 10.1002/adma.202104381

**Published:** 2021-10-10

**Authors:** Jian Qing, Sankaran Ramesh, Qiang Xu, Xiao‐Ke Liu, Heyong Wang, Zhongcheng Yuan, Zhan Chen, Lintao Hou, Tze Chien Sum, Feng Gao

**Affiliations:** ^1^ Siyuan Laboratory Guangzhou Key Laboratory of Vacuum Coating Technologies and New Energy Materials Department of Physics Jinan University Guangzhou Guangdong 510632 P. R. China; ^2^ Department of Physics, Chemistry, and Biology (IFM) Linköping University Linköping SE‐58183 Sweden; ^3^ Division of Physics and Applied Physics School of Physical and Mathematical Sciences Nanyang Technological University 21 Nanyang Link Singapore 637371 Singapore; ^4^ Energy Research Institute @NTU (ERI@N) Interdisciplinary Graduate Programme Nanyang Technological University 50 Nanyang Avenue, S2‐B3a‐01 Singapore 639798 Singapore

**Keywords:** electroluminescence, light‐emitting diodes, pure red, Ruddlesden–Popper perovskites, spacer cation

## Abstract

Perovskite light‐emitting diodes (PeLEDs) have recently shown significant progress with external quantum efficiencies (EQEs) exceeding 20%. However, PeLEDs with pure‐red (620−660 nm) light emission, an essential part for full‐color displays, remain a great challenge. Herein, a general approach of spacer cation alloying is employed in Ruddlesden–Popper perovskites (RPPs) for efficient red PeLEDs with precisely tunable wavelengths. By simply tuning the alloying ratio of dual spacer cations, the thickness distribution of quantum wells in the RPP films can be precisely modulated without deteriorating their charge‐transport ability and energy funneling processes. Consequently, efficient PeLEDs with tunable emissions between pure red (626 nm) and deep red (671 nm) are achieved with peak EQEs up to 11.5%, representing the highest values among RPP‐based pure‐red PeLEDs. This work opens a new route for color tuning, which will spur future developments of pure‐red or even pure‐blue PeLEDs with high performance.

## Graphical abstract

A simple and general strategy of spacer cation alloying is developed to modulate the thickness distribution of quantum wells in Ruddlesden–Popper perovskite films. Based on this method, efficient perovskite light‐emitting diodes with tunable emissions between pure red (626 nm) and deep red (671 nm) are achieved with peak external quantum efficiencies up to 11.5%.

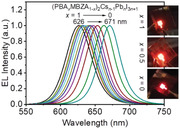

## 1 Introduction

Metal halide perovskites are an emerging new class of emitting materials for efficient light‐emitting diodes (LEDs). This is because they exhibit excellent properties including sharp emission peaks, high photoluminescence quantum yields (PLQYs), tunable bandgaps, and facile solution processability.^[^ ^1^, ^2^, ^3^, ^4^ ^]^ Remarkable progress has been achieved in green, red, and near‐infrared perovskite LEDs (PeLEDs) with external quantum efficiencies (EQEs) exceeding 20%.^[^ ^5^, ^6^, ^7^, ^8^, ^9^, ^10^ ^]^ For full‐color display applications, efficient PeLEDs with high color purity of blue, green, and red are required. Particularly, pure‐red (620−660 nm) emission is imperative according to the color gamut standards for displays; for instance, the Digital Cinema Initiative Primary 3 (DCI‐P3) for digital cinema and the newly defined International Telecommunication Union (ITU) Recommendation BT 2020 (Rec. 2020) for ultra‐high‐definition television call for red emissions with Commission Internationale de l'Eclairage (CIE) chromaticity coordinates of (0.680, 0.320) and (0.708, 0.292), respectively.^[^ ^11^, ^12^, ^13^ ^]^

To achieve the desirable pure‐red PeLEDs, several approaches have been developed to precisely tune the emission wavelengths. One widely employed strategy is mixed bromide‐iodide perovskites, which, however, usually suffer from spectral instability due to halide separation under electrical bias.^[^ ^14^, ^15^, ^16^ ^]^ For instance, although red PeLEDs based on anion‐exchanged CsPbI_3‐_ *_x_* Br*_x_* perovskite quantum dots have demonstrated a high EQE of 21.3%, the electroluminescence (EL) spectra of these PeLEDs showed obvious redshifts and became broader during device operation.^[^ ^7^ ^]^

Another strategy of color tuning for red emission relies on quantum confinement effects in low‐dimensional pure iodide‐based perovskites, especially Ruddlesden–Popper perovskites (RPPs).^[^ ^17^, ^18^ ^]^ Pure iodide‐based 2D/quasi‐2D RPPs have a general formula of (RNH_3_)_2_A*_n_*_‐1_Pb*_n_* I_3_ *_n_*_+1_, where RNH_3_^+^ is a spacer cation, A is a monovalent cation, and *n* is the number of [PbI_6_]^4−^ sheets sandwiched between the spacer layers (which form a “quantum well”, QW).^[^ ^19^, ^20^, ^21^ ^]^ The bandgaps of these RPPs increase with decreasing thicknesses of the QWs, that is, the *n* values. Nevertheless, RPP films prepared following the formula with a nominal *n* value typically consist of multiple QW structures, and cascade energy funneling occurs from thin QWs (large bandgap) to thick ones (small bandgap).^[^ ^22^, ^23^ ^]^ Thus, the emission spectra of RPP films are largely determined by the thicker QWs with smaller bandgaps.

Current approaches for tuning the emission wavelengths of RPP‐based PeLEDs are to substitute the organic spacer cations or to change the nominal *n* values of the precursors. Several organic spacer cations, including 1‐naphthylmethylammonium (NMA),^[^ ^24^ ^]^ benzylammonium (BA),^[^ ^25^ ^]^ and phenylbutylammonium (PBA),^[^ ^26^ ^]^ have been introduced into CsPbI_3_ to form RPPs for red PeLEDs, achieving optimized EQEs of 7.3%, 6.23%, and 13.3%, respectively. However, their emissions are in the deep‐red region (660−700 nm) due to the formation of thick QWs in these RPP films. Further increasing the content of spacer cation can facilitate the formation of thinner QWs, shifting the emission to the pure‐red region; meanwhile, however, this has resulted in PeLEDs with significantly reduced EQEs from 6.23% to 0.41% together with broad multiple EL peaks, which can be attributed to poor charge transport and inefficient energy funneling.^[^ ^25^ ^]^ Therefore, the development of desirable pure‐red PeLEDs with good spectral stability and high efficiency is urgently required yet challenging.

Herein, we demonstrate a simple general approach of spacer cation alloying to precisely tune the emission wavelengths of RPP films for achieving efficient pure‐red PeLEDs. Spacer cations of PBA and α‐methylbenzylammonium (MBZA) are chosen as a representative pair for alloyed (PBA*_x_* MBZA_1‐_ *_x_*)_2_Cs*_n_*_‐1_Pb*_n_* I_3_ *_n_*_+1_ RPP films. (PBA)_2_Cs*_n_*_‐1_Pb*_n_* I_3_ *_n_*_+1_ film possesses narrow distribution of QW thicknesses with short‐wavelength EL peaking at 626 nm, while (MBZA)_2_Cs*_n_*_‐1_Pb*_n_* I_3_ *_n_*_+1_ film shows wide distribution of QW thicknesses with long‐wavelength EL peaking at 671 nm. By simply tuning the alloying ratio of PBA:MBZA, medium distributions of QW thicknesses can be obtained in (PBA*_x_* MBZA_1‐_ *_x_*)_2_Cs*_n_*_‐1_Pb*_n_* I_3_ *_n_*_+1_ films without deteriorating charge‐transporting ability and energy funneling processes. As a result, the alloyed RPP films show continuously tunable emissions between pure red (626 nm) and deep red (671 nm) by adjusting the alloying ratios of PBA:MBZA. Moreover, the resulting PeLEDs show high peak EQEs up to 11.5% with excellent spectral stability; in particular, the PeLEDs with the desirable CIE coordinates of (0.68, 0.32) and (0.71, 0.29) show high peak EQEs of 7.0% and 11.5%, respectively.

## 2 Results and Discussion

### 2.1 Evidence for Spacer Cation Alloying

**Figure 1:**
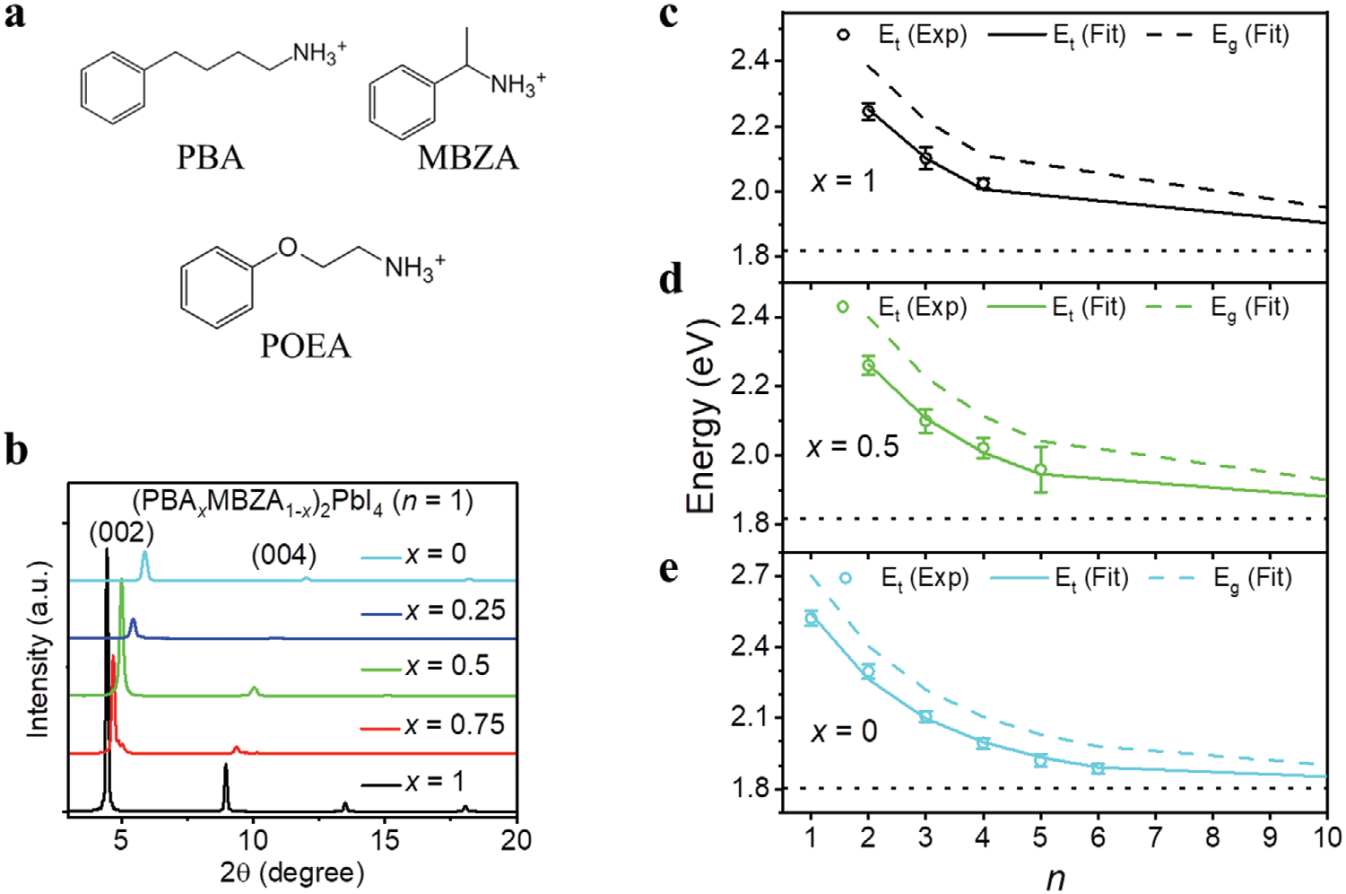
a) Chemical structures of PBA, MBZA, and POEA. b) XRD patterns of *n* = 1 RPP films of (PBA*_x_* MBZA_1‐_ *_x_*)_2_PbI_4_. c–e) Finite potential QW superlattice model to fit resonance energies of *x* = 1 (c), *x* = 0.5 (d), and *x* = 0 (e) samples. Solid lines indicate the fitted energies and dashed lines indicate the fitted band gaps. The black dotted lines indicate the fitted bandgap (≈1.80 eV) of CsPbI_3_.

**Figure** **1**a shows chemical structures of the spacer cations studied in this work: PBA, MBZA, and phenoxyethylammonium (POEA). We prepared *n* = 1 RPP films of (PBA*_x_* MBZA_1‐_ *_x_*)_2_PbI_4_ (*x* = 1, 0.75, 0.5, 0.25, and 0), of which X‐ray diffraction (XRD) patterns are shown in Figure 1b. The diffraction peaks of (PBA)_2_PbI_4_ and (MBZA)_2_PbI_4_ are not found in the alloyed (PBA*_x_* MBZA_1‐_ *_x_*)_2_PbI_4_ (*x* = 0.75, 0.5, 0.25), suggesting the absence of (PBA)_2_PbI_4_ and (MBZA)_2_PbI_4_ in the alloyed RPPs. In addition, all samples exhibit periodical diffraction peaks with different peak positions, indicating the formation of layered structures with different layer distances. According to the Bragg's law, the (002) *d*‐spacing of (PBA*_x_* MBZA_1‐_ *_x_*)_2_PbI_4_ (*x* = 1, 0.75, 0.5, 0.25, and 0) are calculated to be 19.8, 18.8, 17.6, 16.2, and 15.0 Å, respectively, showing gradually decreased layer distances from (PBA)_2_PbI_4_ to (MBZA)_2_PbI_4_. These results indicate that PBA and MBZA can mix uniformly to act as co‐spacer cations in the alloyed RPPs.

To affirm the observation of spacer cation alloying, we employ a periodic QW superlattice model for RPP as described in our previous work.^[^ ^27^ ^]^ The details are given in Supporting Information. Briefly, we approximate the energy landscape along the stacking axis of the RPP as a periodic finite potential QW superlattice structure; the inorganic [PbI_6_]^4−^ octahedra layers form the potential wells, and the organic spacer layers form the potential barriers with a well‐defined width *L_b_* (Figure S1, Supporting Information) as well as effective barrier heights. Changing the organic spacer composition modulates the barrier widths and heights, thereby affecting the optical properties of the system.

Our model accurately fits the exciton resonance energies of the pure (*x* = 0, 1) as well as the alloyed (*x* = 0.5) RPP systems (Figure 1c–e), which accounts for the effects of quantum and dielectric confinement.^[^ ^28^ ^]^ The effective potential barrier heights are also extracted, showing gradual increase from *x* = 1 to *x* = 0 (Table S1, Supporting Information). The result suggests that our model is universally applicable to pure and alloyed systems. The graded values of the potential barrier energy (from model) and *L_b_* (from XRD) imply that in the alloyed film, the barrier layer contains a uniform spacer‐cation alloyed composition rather than being composed of discrete pure phases with different barrier widths.

### 2.2 Precisely Tunable Photoluminescence upon Spacer Cation Alloying

In order to achieve efficient red emission, we prepared higher order RPP films of (PBA*_x_* MBZA_1‐_ *_x_*)_2_Cs*_n_*_‐1_Pb*_n_* I_3_ *_n_*_+1_ (*x* = 1, 0.75, 0.5, 0.25, and 0), where *n* ≈ 2 based on the loading molar ratios of the precursors. The molecule 2,2'‐[oxybis(ethylenoxy)]diethylamine (ODEA) was added into precursor solutions for defect passivation (See Experimental Section for details).^[^ ^8^ ^]^ Surface morphologies of the RPP films are investigated by scanning electron microscopy (SEM) and atomic force microscopy (AFM) (Figures S2 and S3, Supporting Information). These films all show pinhole‐free smooth morphologies with very low root‐mean‐squared roughness (around 0.5 nm in an area of 5 µm × 5 µm), which helps to avoid leakage current in devices. XRD results in Figure S4, Supporting Information, reveal the layered structures of the RPP films.^[^ ^25^ ^]^

**Figure 2:**
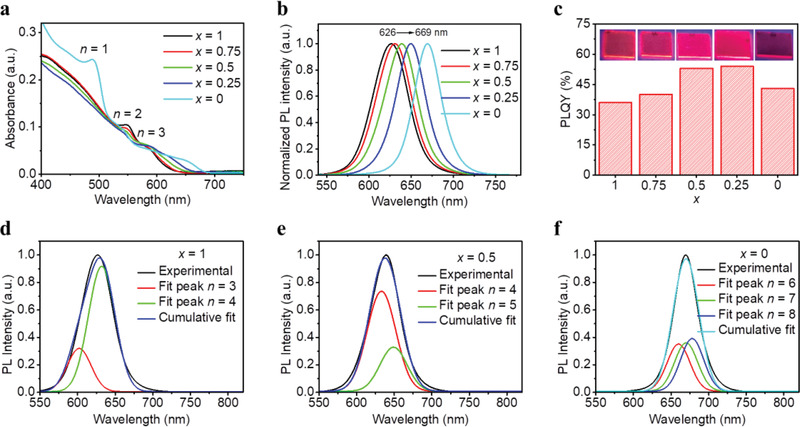
a) Absorption spectra, b) normalized PL spectra, and c) PLQYs of (PBA*_x_* MBZA_1‐_ *_x_*)_2_Cs*_n_*_‐1_Pb*_n_* I_3_ *_n_*_+1_ RPP films. The inset is photos of the corresponding RPP films under 365 nm UV light. d–f) Gaussian peak fitting results of the PL spectra for the (PBA*_x_* MBZA_1‐_ *_x_*)_2_Cs*_n_*_‐1_Pb*_n_* I_3_ *_n_*_+1_ RPP films with *x* = 1 (d), *x* = 0.5 (e), and *x* = 0 (f).

**Figure** **2**a shows ultraviolet–visible (UV–vis) absorption spectra of the RPP films. Multiple excitonic absorption peaks at around 490, 550, and 590 nm are observed, corresponding to the QWs with *n* = 1, 2, and 3, respectively. This is consistent with previous reports, confirming that the films contain mixed QW structures.^[^ ^22^, ^26^, ^29^ ^]^ Interestingly, a stepwise monotonous redshift of absorption edges is observed with decreasing *x* value. This observation suggests that the overall bandgaps of these RPP films decrease with increasing MBZA content.

The corresponding PL spectra in Figure 2b show a gradual redshift with PL peaks from 626 nm (*x* = 1) to 669 nm (*x* = 0), which agrees well with the shift of absorption edges. In addition, all the films show absent or weak emission peaks from *n* = 2 and 3 QWs, implying efficient energy funneling from small‐*n* to large‐*n* QWs (Figure S5a, Supporting Information). The PLQYs of these RPP films and their photographs under UV light (365 nm) are given in Figure 2c, showing that all films have quite strong red PL emissions with high PLQYs over 36%. A maximum PLQY of 54% is obtained for the sample with *x* = 0.25. Compared to pure PBA‐ and pure MBZA‐based RPP films, the higher PLQYs of the PBA:MBZA‐alloyed films may be explained by synergistic effect of dual spacer cations to suppress nonradiative recombination.^[^ ^30^, ^31^, ^32^, ^33^ ^]^ Moreover, by carefully tuning the alloying ratio of PBA:MBZA, we can precisely modulate the emission peaks between 626 and 669 nm (Figure S5b, Supporting Information).

### 2.3 Mechanism of Precisely Tunable Photoluminescence

To understand the mechanism behind the precisely tunable PL upon spacer cation alloying, we focus on the emissive centers of the RPP films. We speculate that the PL spectra of the RPP films are attributed to multiple emissions of different QWs rather than a single QW. To verify this point, we first calculate the PL peaks of CsPbI_3_ based RPP QWs with different *n* values based on a model using the modified effective mass approximation.^[^ ^26^, ^34^ ^]^ The results are shown in Table S2, Supporting Information; for instance, the RPP QWs with *n* = 2, 3, and 4 show PL peaks at 569, 608, and 632 nm, respectively.

We then fabricated RPP films with obvious multiple PL peaks by adjusting the molar ratios of PBAI:CsI:PbI_2_ (2:3:2 and 2:2.6:2, respectively) in the precursor solutions. The PL spectra of the two films are shown in Figure S6, Supporting Information, which can be well fitted by multiple‐peak fitting with Gaussian function.^[^ ^35^ ^]^ Both films exhibit the same fitted PL peaks at ≈562, ≈600, and ≈633 nm though with different intensities. The fitted PL peaks are in good agreement with the calculated ones (Table S2, Supporting Information), suggesting that the PL spectra of the two films are a combination of emissions from QWs with *n* = 2, 3, and 4.

Similarly, we infer that the PL spectra of the (PBA*_x_* MBZA_1‐_ *_x_*)_2_Cs*_n_*_‐1_Pb*_n_* I_3_ *_n_*_+1_ RPP films are a combination of emissions from several large‐*n* QWs despite single PL peaks (Figure 2b). As shown in Figure 2d, the PL spectrum of the *x* = 1 based RPP film can be well fitted with PL peaks of *n* = 3 and *n* = 4 QWs using Gaussian function. Meanwhile, the PL spectra of the *x* = 0.5 and 0 based films can be well fitted with PL peaks of *n* = 4, 5 QWs and *n* = 6, 7, 8 QWs, respectively (Figure 2e,f). The fitting results prove that the PL spectra of the RPP films can be assigned to multiple emissions of several large‐*n* RPP QWs with *n* in the range of 3−8. These results also suggest that the emissive large‐*n* QWs of the (PBA*_x_* MBZA_1‐_ *_x_*)_2_Cs*_n_*_‐1_Pb*_n_* I_3_ *_n_*_+1_ RPP films gradually become thicker with increasing MBZA content.

Further, we infer that pure PBA and pure MBZA can enable the formation of RPP films with relatively narrow and wide distributions of QW thicknesses (or *n* values), respectively, and medium distributions of QW thicknesses can be obtained by tuning the alloying ratio of PBA:MBZA. Since the nominal *n* values (≈ 2) as suggested by the loading molar ratios of the precursors are the same for all RPP films, the average QW thicknesses (or *n* values) should be the same. As discussed above, an increasing amount of MBZA results in thicker emissive QWs, the thinner non‐emissive QWs in these films should hence be in larger proportion to maintain the same average QW thickness. Indeed, the MBZA‐based film shows obvious excitonic absorption peak of *n* = 1 QW (Figure 2a) and PL peaks of *n* = 1 and 2 QWs (Figure S5a, Supporting Information).

Based on the results above, we can reasonably elucidate the mechanism of the precisely tunable PL upon spacer cation alloying: 1) the PL spectra of RPP films are a combination of emissions from several large‐*n* QWs; 2) PBA enables the formation of RPP films with narrow QW thickness distribution and thin QWs, showing short‐wavelength emission. With increasing MBZA content in the alloyed RPP films, the distribution of QW thicknesses gradually becomes wider with thicker QWs, consequently resulting in gradually redshifted PL spectra.

### 2.4 Transient Absorption Spectroscopy

**Figure 3:**
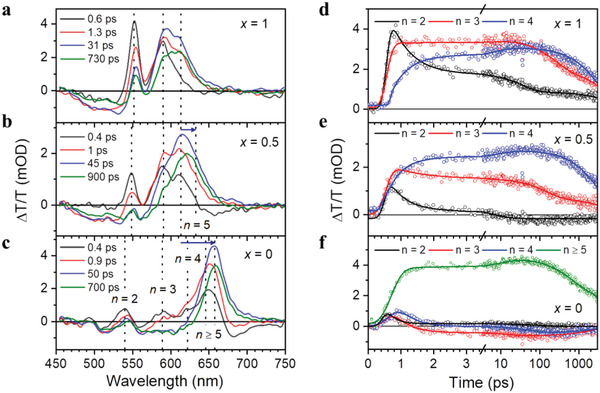
a–c) Slices of TA spectra for the (PBA*_x_* MBZA_1‐_ *_x_*)_2_Cs*_n_*_‐1_Pb*_n_* I_3_ *_n_*_+1_ RPP films with *x* = 1 (a), *x* = 0.5 (b), and *x* = 0 (c). Dotted vertical lines indicate the photobleaching (PB) of *n*‐thickness QWs. Blue horizontal arrows in (b,c) indicate the redshift of the dominant PB peaks toward larger‐*n* QWs. d–f) Kinetics of PB peaks for the RPP films with *x* = 1 (d), *x* = 0.5 (e), *x* = 0 (f) with multi‐exponential fits. The olive data in (f) corresponds to *n* ≥ 5 PB at 652 nm. Details of lifetime fitting of kinetics can be found in Table S3, Supporting Information.

To gain more insights into the effect of spacer cation alloying on the QW distribution and energy funneling process in the RPP films, we conducted transient absorption (TA) measurements (**Figure** **3** and Figure S7, Supporting Information). Three distinctive ground‐state photobleaching (PB) peaks are observed in all samples at ≈550, ≈590, and ≈615 nm, corresponding to *n* = 2, 3, and 4 QWs, respectively, with the PB energies shifting slightly depending on the spacer cations (Figure 3a–c). Notably, additional PB peaks at longer wavelengths corresponding to *n* = 5 and *n* ≥ 5 QWs appear in the RPP films with *x* = 0.5 and 0, respectively. This results in a redshift of the dominant PB peak to longer wavelengths at later delay time, which is shown by blue horizontal arrows in Figure 3b,c. These results confirm that the distributions of QW thicknesses in the RPP films gradually become wider with the increase of MBZA ratio, in good agreement with the observation by absorption and PL spectra.

The TA kinetics are further analyzed in Figure 3d–f. The assignment of fitted lifetime components are given in the Supporting Information and results of lifetime fitting are detailed in Table S3, Supporting Information. Ultrafast funneling of carriers from small‐*n* to large‐*n* QWs within the first few picoseconds after photoexcitation is observed in all films. In the *x* = 1 film (Figure 3d), the *n* = 2 PB shows ultrafast decay, acting as carrier “donor” QW, while those corresponding to *n* = 3, 4 grow simultaneously within tens of picoseconds after photoexcitation, making them carrier “acceptor” QWs. Analysis of the early‐time kinetics of the *x* = 0.5 sample (Figure 3e) and fitted lifetime values (Table S3, Supporting Information) show that both the *n* = 2, 3 QWs are donors while *n* = 4, 5 QWs are acceptors. For the *x* = 0 sample (Figure 3c), PB of a wider distribution of larger, overlapping *n* ≥ 5 QWs is observed at ≈652 nm, along with additional peak corresponding to *n* = 1 QW at ≈490 nm. Analysis of fitted kinetics of funneling in Figure 3f shows that *n* = 2, 3, 4 QWs are all donor QWs with rapidly decaying populations while *n* ≥ 5 QWs are acceptors. The “acceptor” QWs in each sample corresponds to the emissive QWs estimated from the fitting of PL spectra, shown earlier in Figure 2d–f. This approach of spacer cation alloying in the RPP system bears similarity to color‐tunable emission achieved via donor–acceptor energy transfer dyads,^[^ ^36^, ^37^ ^]^ where the emission wavelength is modulated by changing the acceptor molecule. In our approach, however, this is achieved by simply tuning the alloying ratio of spacer cations, and different‐*n* RPP QWs acting as donor and acceptor.

### 2.5 Device Performance of PeLEDs

To explore EL properties of (PBA*_x_* MBZA_1‐_ *_x_*)_2_Cs*_n_*_‐1_Pb*_n_* I_3_ *_n_*_+1_ RPP films, we fabricated and optimized PeLEDs with a device architecture of indium tin oxide (ITO)/poly(3,4‐ethylenedioxythiophene) polystyrene sulfonate (PEDOT:PSS)/poly(9,9‐dioctyl‐fuorene‐*co*‐N‐(4‐butylphenyl)diphenylamine) (TFB)/poly(9‐vinlycarbazole) (PVK)/RPP film/1,3,5‐tris(1‐phenyl‐1H‐benzimidazol‐2‐yl)benzene (TPBi)/lithium fluoride (LiF)/Al. A trilayer structure of PEDOT:PSS/TFB/PVK and TPBi film are used as hole‐injection and electron‐injection layers, respectively. The thicknesses of RPP films with different alloying ratios of PBA:MBZA are around 40 nm. A clear sandwich structure can be observed from a cross‐sectional SEM image of a typical device (Figure S8a, Supporting Information). A schematic of energy level diagram is shown in Figure S8b, Supporting Information.

**Figure 4:**
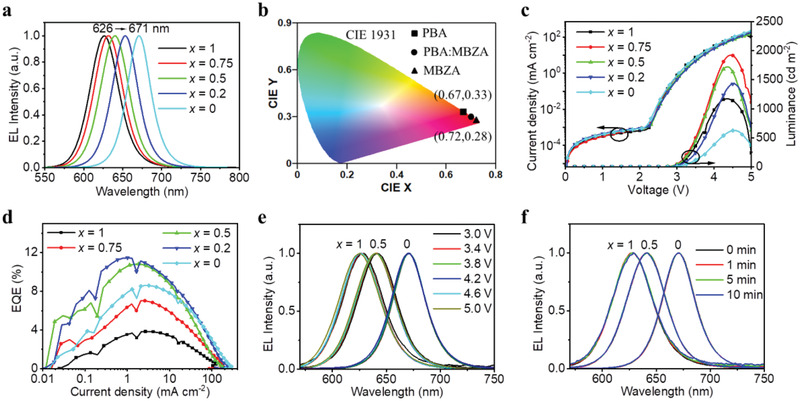
a) Normalized EL spectra, b) CIE 1931 chromatic coordinates, c) current density–voltage–luminance characteristics, and d) EQE values versus current density of the PeLEDs. e) Normalized EL spectra of PeLEDs under various bias voltages with *x* = 1, 0.5, and 0. f) Normalized EL spectra of PeLEDs under a constant bias voltage of 3.2 V with *x* = 1, 0.5, and 0.

We summarize the device performance in **Figure** **4**, **Table** **1**, and Figure S9, Supporting Information. EL spectra of these PeLEDs show a gradual redshift of EL peaks from 626 to 671 nm with increasing MBZA content (Figure 4a), which is in good agreement with the change of PL spectra. All the EL emissions exhibit only one dominant peak with narrow full‐width at half‐maximum (FWHM) of 36–43 nm, resulting in high color purity in CIE coordinates from pure red to deep red (Figure 4b). Particularly, the emissions for PeLEDs with *x* = 0.75 and 0.2 reach the DCI‐P3 (0.680, 0.320) and Rec. 2020 standards (0.708, 0.292) for the red color, respectively. Although the color purity of these PeLEDs has already met the high standards, their EL spectra may be further narrowed, for instance, by improving phase purity of the RPP films and enhancing the energy funneling process.^[^ ^38^, ^39^ ^]^

**Table 1:** The performance parameters of PeLEDs based on PBA:MBZA‐alloyed RPP films with different alloying ratios (*x*)

RPP films	Peak EQE [%]	Peak current efficiency [cd A^−1^]	Maximum luminance [cd m^−2^]	EL peak [nm]	FWHM [nm]	CIE (*x*, *y*)
*x* = 1	3.8	5.2	1173	626	43	(0.67, 0.33)
*x* = 0.75	7.0	8.0	1927	632	43	(0.68, 0.32)
*x* = 0.5	10.8	9.4	1724	640	42	(0.69, 0.31)
*x* = 0.2	11.5	5.7	1431	653	39	(0.71, 0.29)
*x* = 0	8.6	1.7	626	671	36	(0.72, 0.28)

The current density−voltage (*J*−*V*) curves show no obvious difference for the PeLEDs with different alloying ratios of PBA:MBZA (Figure 4c), suggesting similar charge injection and transport ability of the RPP films. In general, charge injection and transport ability of RPP films is predominantly determined by the loading amount of organic spacer cations, and the higher the amount of organic spacer cations, the poorer the charge injection and transport of RPP films.^[^ ^23^, ^40^, ^41^ ^]^ The unchanged total amount of organic spacer cations in our approach enables wavelength‐tuning of RPPs (Figure 4a) without deteriorating their charge injection and transport ability (Figure 4c). Moreover, as shown in the energy level diagram of our device (Figure S8b, Supporting Information), the deep HOMO (highest occupied molecular orbital) of PVK and shallow LUMO (lowest unoccupied molecular orbital) of TPBi allow efficient hole and electron injection even into the largest‐bandgap QWs, respectively.

Maximum luminance between 600 and 2000 cd m^−2^ are achieved (Figure 4c), which are comparable to previous studies.^[^ ^25^, ^26^ ^]^ The peak EQEs increase first from 3.8% to ≈11.0%, and then decrease to 8.6% with increasing MBZA content (Figure 4d and Figure S9c, Supporting Information), which are consistent with the PLQY measurements. The highest peak EQE of 11.5% is achieved for the PeLEDs with *x* = 0.2 (Table 1). Notably, to the best of our knowledge, the peak EQEs for pure‐red emissions between 626 and 650 nm represent the best for the RPP‐based PeLEDs (Table S4 and S5, Supporting Information).

In addition, histograms of peak EQEs show average values of 3.6%, 10.2%, and 8.3% with relative standard deviations of 9.8%, 5.5%, and 8.0% for PeLEDs with *x* = 1, 0.5, and 0, respectively, indicating good reproducibility of our high‐efficiency red PeLEDs (Figure S10, Supporting Information). Photos of working PeLEDs are presented in Figure S11, Supporting Information, which show bright red‐light emission. In contrast to the method of changing the ratios of precursor materials for color tuning, which deteriorates the device performance,^[^ ^25^ ^]^ the method of spacer cation alloying even increases the peak EQEs. This could be due to the fact that spacer cation alloying maintains the good charge‐transport ability and efficient energy funneling in the RPP films.

We then study the spectral and operational stability of the PeLEDs. With bias voltage increased from 3 to 5 V, the EL spectra show negligible shifts (<2 nm) (Figure 4e). In addition, the normalized EL spectra of the PeLEDs remain unchanged under continuous operation at 3.2 V and at maximum luminance (Figure 4f and Figure S12a, Supporting Information). These results demonstrate the excellent spectral stability of the RPP‐based PeLEDs. Figure S12c, Supporting Information, presents the lifetime measurements at a constant current density with an initial luminance of 100 cd m^−2^. The half‐lifetimes of the PeLEDs with *x* = 1, 0.5, and 0 are 0.9, 25, and 21 min, respectively, which are far from the values for commercial purpose. Note that the poor long‐term stability of PeLEDs is a common issue at present, which requires further investigations.^[^ ^42^, ^43^ ^]^

### 2.6 General Applicability of Spacer Cation Alloying Approach

**Figure 5:**
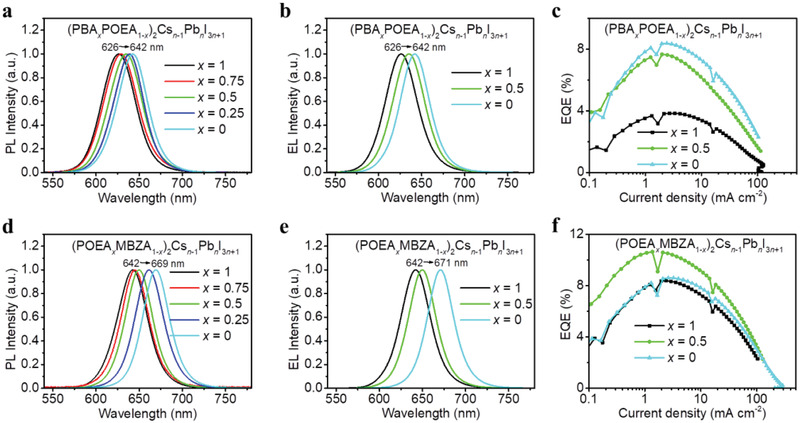
a) Normalized PL spectra, b) EL spectra, and c) EQE values versus current density of (PBA*_x_* POEA_1‐_ *_x_*)_2_Cs*_n_*_‐1_Pb*_n_* I_3_ *_n_*_+1_ RPP films. d) Normalized PL spectra, e) EL spectra, and f) EQE values versus current density of (POEA*_x_* MBZA_1‐_ *_x_*)_2_Cs*_n_*_‐1_Pb*_n_* I_3_ *_n_*_+1_ RPP films.

To verify the general applicability of the spacer cation alloying approach, we investigate another two spacer cation pairs of PBA:POEA and POEA:MBZA as further examples. POEA‐based PeLEDs also show good performance with a peak EQE of 8.4% and EL peak at 642 nm. Similarly, with increasing POEA content, (PBA*_x_* POEA_1‐_ *_x_*)_2_Cs*_n_*_‐1_Pb*_n_* I_3_ *_n_*_+1_ RPP films show gradually tuned emissions from 626 to 642 nm (**Figure** **5**a,b) and the resulting PeLEDs achieve peak EQEs from 3.8% to 8.4% (Figure 5c). With increasing MBZA content, (POEA*_x_* MBZA_1‐_ *_x_*)_2_Cs*_n_*_‐1_Pb*_n_* I_3_ *_n_*_+1_ RPP films show gradually tuned emissions from 642 to 671 nm (Figure 5d,e) and the resulting PeLEDs show good device performance with peak EQEs up to 11.0% (Figure 5f).

We note that the performance of PeLEDs based on alloyed spacer cations is limited by the spacer cation with lower performance, which is PBA for this work. Therefore, further studies on spacer cations are anticipated to produce highly efficient PeLEDs with pure red or deep red emissions, and then medium emissions with high performance can be achieved by simply tuning the alloying ratio of the spacer cations. Furthermore, this method is expected to be effective in fabricating pure‐blue PeLEDs by replacing I with Br in the precursor solutions, which is currently under investigation.

## 3 Conclusion

In summary, we have demonstrated a general strategy of spacer cation alloying for efficient red PeLEDs with precisely tunable wavelengths. By simply tuning the alloying ratio of PBA:MBZA, the distribution of QW thicknesses in the alloyed (PBA*_x_* MBZA_1‐_ *_x_*)_2_Cs*_n_*_‐1_Pb*_n_* I_3_ *_n_*_+1_ RPP films can be precisely modulated without deteriorating the charge‐transport ability and energy funneling processes. The resulting PeLEDs show continuously tunable emissions between pure red (626 nm) and deep red (671 nm) with high peak EQEs up to 11.5% and excellent spectral stability. Notably, the peak EQEs for pure‐red emissions between 626 and 650 nm represent the best for the RPP‐based PeLEDs. Moreover, the effect of spacer cation alloying on the properties of RPP films is generalized to other spacer cations. Our work provides useful guidelines for achieving efficient and wavelength‐tunable PeLEDs.

## 4 Experimental Section

### Materials

Poly(3,4‐ethylenedioxythiophene):poly(styrenesulfonate) (PEDOT:PSS, AI 4083) was purchased from Heraeus. Poly(9,9‐dioctyl‐fuorene‐*co*‐N‐(4‐butylphenyl)diphenylamine) (TFB) was purchased from Ossila. PbI_2_ (beads, 99.999%) was purchased from Alfa Aesar. 1,3,5‐Tris(1‐phenyl‐1H‐benzimidazol‐2‐yl)benzene (TPBi) was purchased from Lumtec. Other chemicals were obtained from Sigma‐Aldrich.

### Synthesis of Organic Ammonium Salt

PBAI was synthesized by adding excess hydroiodic acid (45 wt% in water) to a stirring solution of 4‐phenylbutylamine (PBNH_2_, 4 mmol) in ethanol (5 mL) at 0 °C for 2 h. Then, 60 mL diethyl ether was added into the mixture to obtain PBAI precipitate, which was washed three times with diethyl ether and then dried under vacuum. MBZAI and POEAI were synthesized by a similar method.

### Perovskite Precursor Solutions

The PBA‐based precursor solution was prepared by dissolving PBAI, CsI, PbI_2_, and ODEA with a molar ratio of 1:0.6:1:0.1 in DMF (the concentration of Pb^2+^ is 0.08 m). The MBZA‐based precursor solution was prepared similarly with PBAI substituted by MBZAI. The above two precursor solutions were mixed with different ratios to obtain precursor solutions based on alloyed spacer cations.

### Device Fabrication

Indium tin oxide (ITO) glass substrates were cleaned in aqueous detergent solution before 15 min UV–ozone treatment. PEDOT:PSS was spin‐coated onto the ITO substrates at 4000 rpm, followed by drying at 140 °C for 10 min in air. Then, the samples were transferred into a N_2_‐filled glovebox. TFB (4 mg mL^−1^ in CB) was spin‐coated at 6000 rpm, followed by annealing at 130 °C for 10 min. PVK (4 mg mL^−1^ in CB) was deposited on the TFB film at 2000 rpm, followed by annealing at 130 °C for 10 min. RPP films were fabricated by spin‐coating the precursor solutions at 4000 rpm, followed by annealing on a preheated hotplate at 80 °C for 15 min. Finally, TPBi (35 nm), LiF (1 nm), and Al (100 nm) were deposited by thermal evaporation with vacuum pressure below 4 × 10^−6^ mbar. The active device area was defined as 0.0725 cm^2^ by the overlapping area of Al and ITO electrodes.

### Perovskite Film and Device Characterizations

A scanning electron microscope (SEM, Philips XL30 FEG SEM) and a tapping‐mode atomic force microscope (AFM, Park XE7) were used to characterize the morphology of samples. XRD measurements were performed using an X‐ray diffractometer (Panalytical X’ Pert Pro) with Cu Kα radiation. UV–vis absorption spectra were obtained with a PerkinElmer Lambda 900 in transmission mode. Steady‐state PL spectra were obtained with a 450 nm excitation laser and an Andor spectrometer (Shamrock sr‐303i‐B, coupled to a Newton EMCCD Si array detector). PLQY results of RPP films were obtained by an integrated system of a 450 nm laser, optical fiber, spectrometer, and an integrating sphere. All characterizations of PeLEDs were carried out at room temperature in a N_2_‐filled glovebox. A Keithley 2400 source meter and a fiber integration sphere (FOIS‐1) coupled with a QE Pro spectrometer (Ocean Optics) were used for the measurements. The applied voltage started from 0 V and increased with a step of 0.05 V, lasting for 300 ms at each voltage step for stabilization and test. The integrating sphere‐spectrometer system was calibrated by Vis‐NIR radiometric calibration sources for absolute light intensity and a HG‐1 calibration source for wavelength (Ocean Optics). Lifetime measurements of PeLEDs were conducted using the same testing system.

### Transient Absorption (TA) Spectroscopy

TA measurements were performed with HELIOS spectrometer (Ultrafast Systems LLC). A mode‐locked Ti‐Sapphire oscillator (Coherent Vitesse, 80 MHz) was used as the seed laser to a 1 kHz regenerative amplifier (Coherent Legend) to generate pump pulses of 150 fs width at 800 nm wavelength. The 800 nm wavelength pulse was frequency‐doubled to 400 nm by second harmonic generation (SHG) via a beta‐barium borate (BBO) crystal. Pump fluence used for the study is < 30 µJ cm^‐2^ to keep the excited carrier densities low to minimize multi‐photon excitation and Auger effects. The white‐light continuum probe was generated by focusing a small fraction of the 800 nm pulses on a 2 mm sapphire crystal to and passed through a 750 nm short‐pass filter, to generate the broadband probe light of wavelength 450 to 750 nm.

## Conflict of Interest

The authors declare no conflict of interest.

## Supporting information

Supporting Information

## Data Availability

The data that support our findings and all the codes used in the computation of results in this work are openly available in DR‐NTU (Data) at https://doi.org/10.21979/N9/ORJLUR. Data and codes are also available from the corresponding authors upon reasonable request.
