# Supplementary material for: Spacer Cation Alloying in Ruddlesden–Popper Perovskites for Efficient Red Light‐Emitting Diodes with Precisely Tunable Wavelengths
Source: Adv Mater. 2021 Oct 10;33(49):2104381. doi: 10.1002/adma.202104381 (PMC11468992; doi:10.1002/adma.202104381)
Supplement: Supplementary file 1 — Supporting Information [file ADMA-33-2104381-s001.pdf]

© 2021 Wiley-VCH GmbH

ADVANCED MATERIALS

Supporting Information

for *Adv. Mater.,* DOI: 10.1002/adma.202104381

Spacer Cation Alloying in Ruddlesden–Popper Perovskites for Efficient Red Light-Emitting Diodes with Precisely Tunable Wavelengths

Jian Qing, Sankaran Ramesh, Qiang Xu, Xiao-Ke Liu, Heyong Wang, Zhongcheng Yuan, Zhan Chen, Lintao Hou, Tze Chien Sum,* and Feng Gao**

Supporting Information

Spacer Cation Alloying in Ruddlesden-Popper Perovskites for Efficient Red Light-Emitting Diodes with Precisely Tunable Wavelengths

Jian Qing, Sankaran Ramesh, Qiang Xu, Xiao-Ke Liu, Heyong Wang, Zhongcheng Yuan, Zhan Chen, Lintao Hou, Tze Chien Sum* and Feng Gao**

Dr. J. Qing, Dr. Z. Chen, Prof. L. Hou
Siyuan Laboratory, Guangzhou Key Laboratory of Vacuum Coating Technologies and New Energy Materials, Department of Physics, Jinan University, Guangzhou, Guangdong 510632, P. R. China

Dr. J. Qing, Dr. X.-K. Liu, Dr. H. Wang, Dr. Z. Yuan, Dr. Z. Chen, Prof. F. Gao
Department of Physics, Chemistry, and Biology (IFM), Linköping University, Linköping SE-58183, Sweden
E-mail: xiaoke.liu@liu.se; feng.gao@liu.se

S. Ramesh, Dr. Q. Xu, Prof. T. C. Sum
Division of Physics and Applied Physics, School of Physical and Mathematical Sciences, Nanyang Technological University, 21 Nanyang Link, Singapore 637371, Singapore
E-mail: tzechien@ntu.edu.sg

S. Ramesh
Energy Research Institute @NTU (ERI@N), Interdisciplinary Graduate Programme, Nanyang Technological University, 50 Nanyang Avenue, S2-B3a-01, Singapore 639798, Singapore

Periodic QW superlattice model for RPP

The RPP structure is modeled as an effective finite potential QW superlattice structure with repeating units of inorganic (well) and organic (barrier) layers as shown in Figure S1. The spacer molecule forms the energy barrier for the electron and hole.

The exciton resonance energies E_t for the RPP are expressed as:

$$E_t = E_{\mathrm{G}}^{\mathrm{3D}} + \left\langle \psi_{\mathrm{e}}^{(0)} \middle| H_{\mathrm{e}} \middle| \psi_{\mathrm{e}}^{(0)} \right\rangle + \left\langle \psi_{\mathrm{h}}^{(0)} \middle| H_{\mathrm{h}} \middle| \psi_{\mathrm{h}}^{(0)} \right\rangle - E_{\mathrm{b}} \qquad \text{(S1)}$$

Where E_G^{3D} is the bandgap of the three-dimensional (3D) $CsPbI_3$ phase, and E_b is the exciton binding energy. E_b is calculated assuming a trial 1s wavefunction of two-dimensional hydrogen atom for the exciton. The coulomb interaction between electron and hole is estimated in the presence of dielectric confinement, using the image charge effect.[1] The wavefunctions of the electron and hole in the system are estimated assuming periodic

boundary conditions of the superlattice:

$$\psi(z) = \psi(z + L_w + L_b) \quad \text{(S2)}$$

Where L_w and L_b are the widths of the potential well and barrier, respectively.

Fitting our model to the exciton resonance energies yields the confinement energies of charge carriers, from which the effective electron (V_e^0) and hole barrier heights (V_h^0) are extracted. A complete description of the model can be found in our previous work,[2] where the model was used to predict the excitonic properties of the methylammonium lead halide RPP system. For the cesium lead iodide RPP systems in this work, the inorganic lattice spacing and effective masses of holes and electrons are taken from literature.[3] The organic layer spacing is obtained from the *d*-spacing extracted from the XRD data.

The exciton resonance energies for the $x = 0$, 0.5, 1 RPP films are estimated by fitting the early time TA spectra with gaussian peaks. The extracted resonance energies are then fitted with the model. The results of the fitting are shown in the main text Figure 1c-e and Table S1. The average extracted 3D $CsPbI_3$ band gap is 1.81 eV, which is close to the experimentally reported value.

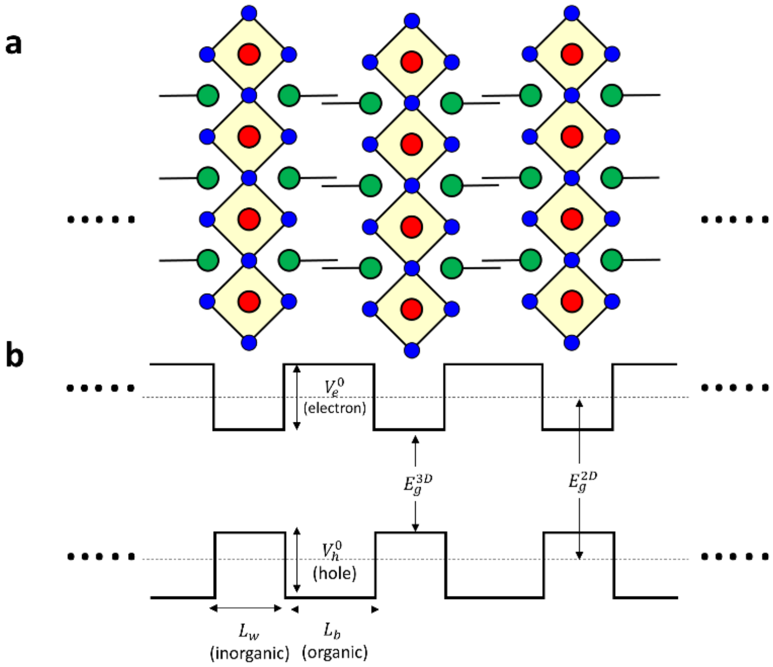

Figure S1. Finite potential QW Model. a) Schematic of the two-dimensional RPP structure with alternating inorganic and organic layers. b) Effective energy landscape along the stacking axis of RPP.

Table S1. MQW model extracted parameters.

RPP (x)	Electron Barrier V_e^0 (eV)	Hole Barrier V_h^0 (eV)	3D bandgap E_g^{3D} (eV)	Barrier Width L_b (nm) (From XRD)
$x = 1$	0.70 ± 0.05	0.44 ± 0.05	1.82 ± 0.05	1.98
$x = 0.5$	0.75 ± 0.05	0.48 ± 0.05	1.82 ± 0.05	1.76
$x = 0$	0.83 ± 0.05	0.52 ± 0.05	1.80 ± 0.05	1.50

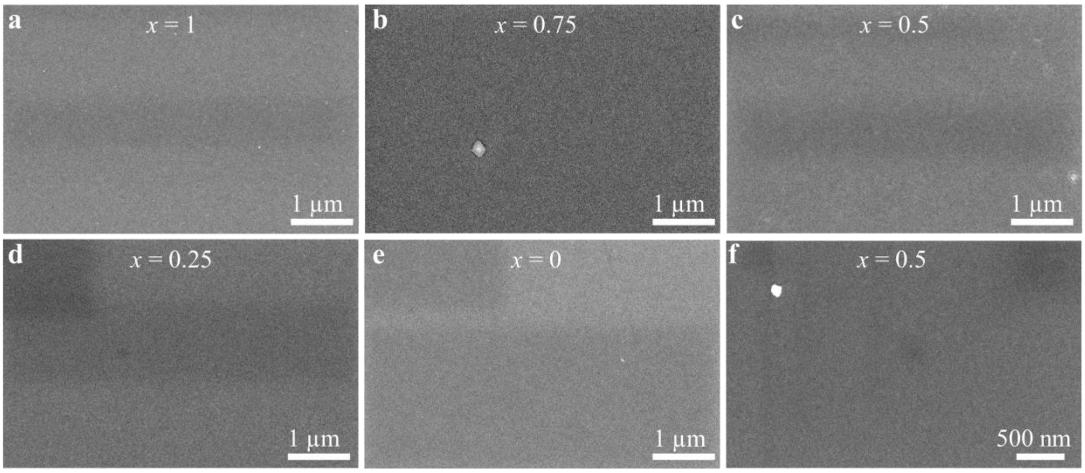

Figure S2. SEM images of the $(PBA_xMBZA_{1-x})_2Cs_{n-1}Pb_nI_{3n+1}$ RPP films with x = a) 1, b) 0.75, c) 0.5, d) 0.25, and e) 0. f) An enlarged SEM image of x = 0.5 sample.

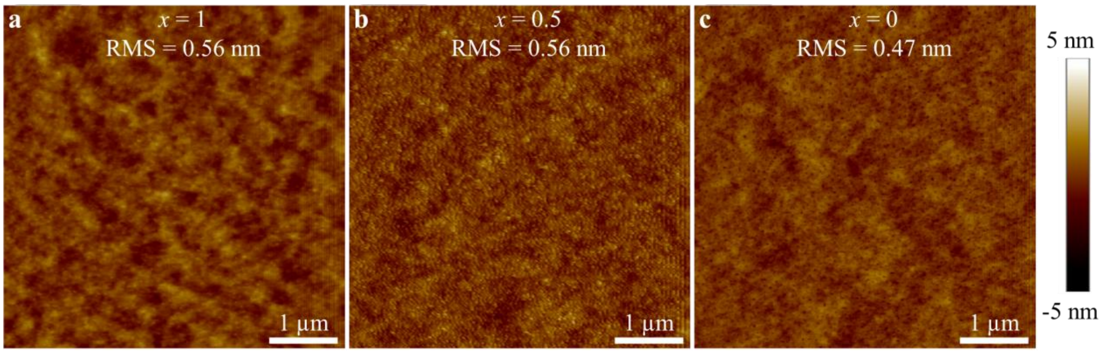

Figure S3. AFM surface images (size: 5 μm × 5 μm) of the $(PBA_xMBZA_{1-x})_2Cs_{n-1}Pb_nI_{3n+1}$ RPP films with x = a) 1, b) 0.5, and c) 0.

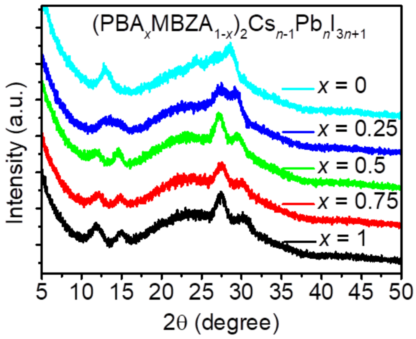

Figure S4. XRD patterns of the $(PBA_xMBZA_{1-x})_2Cs_{n-1}Pb_nI_{3n+1}$ RPP films on glass substrates.

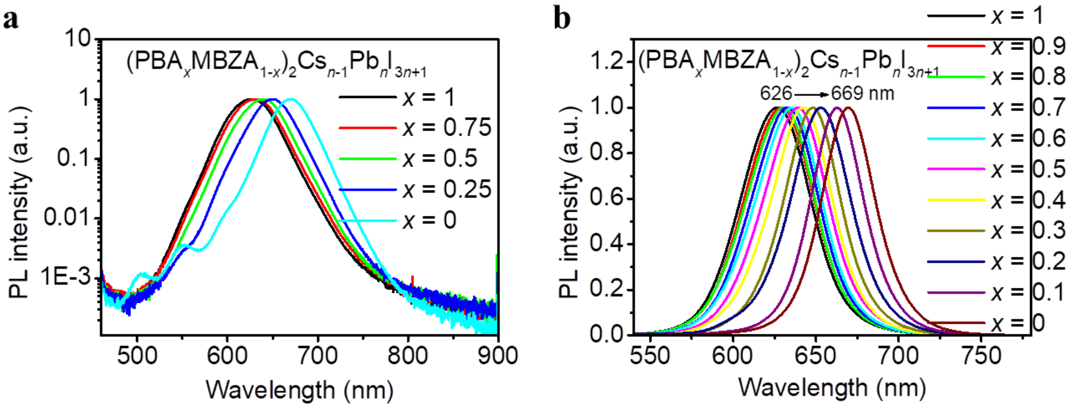

Figure S5. a) PL spectra in log scale and b) PL spectra of the $(PBA_xMBZA_{1-x})_2Cs_{n-1}Pb_nI_{3n+1}$ RPP films with different alloying ratios of PBA:MBZA.

Table S2. Calculated PL peaks of $CsPbI_3$-based RPP QWs with different n values.

n	2	3	4	5	6	7	8	9
PL peak (nm)	569	608	632	648	660	669	676	681

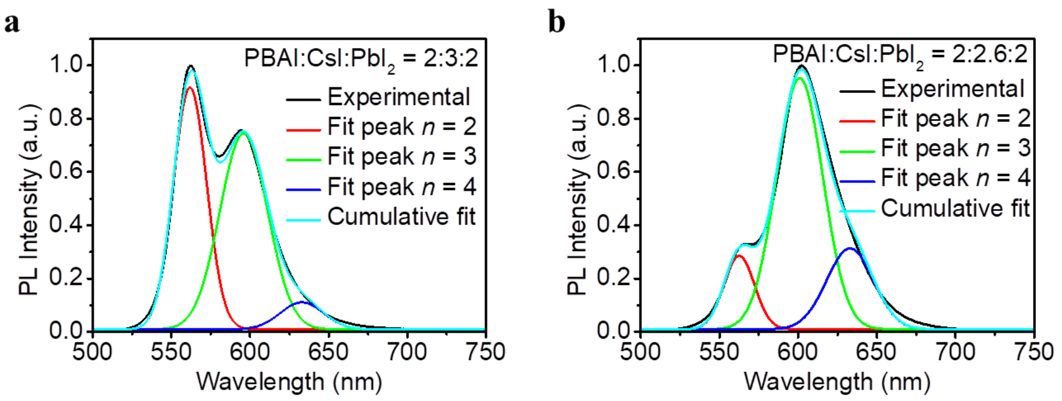

Figure S6. Gaussian peak fitting results of the PL spectra from precursor solutions with $PBAI:CsI:PbI_2$ molar ratios of a) 2:3:2 and b) 2:2.6:2.

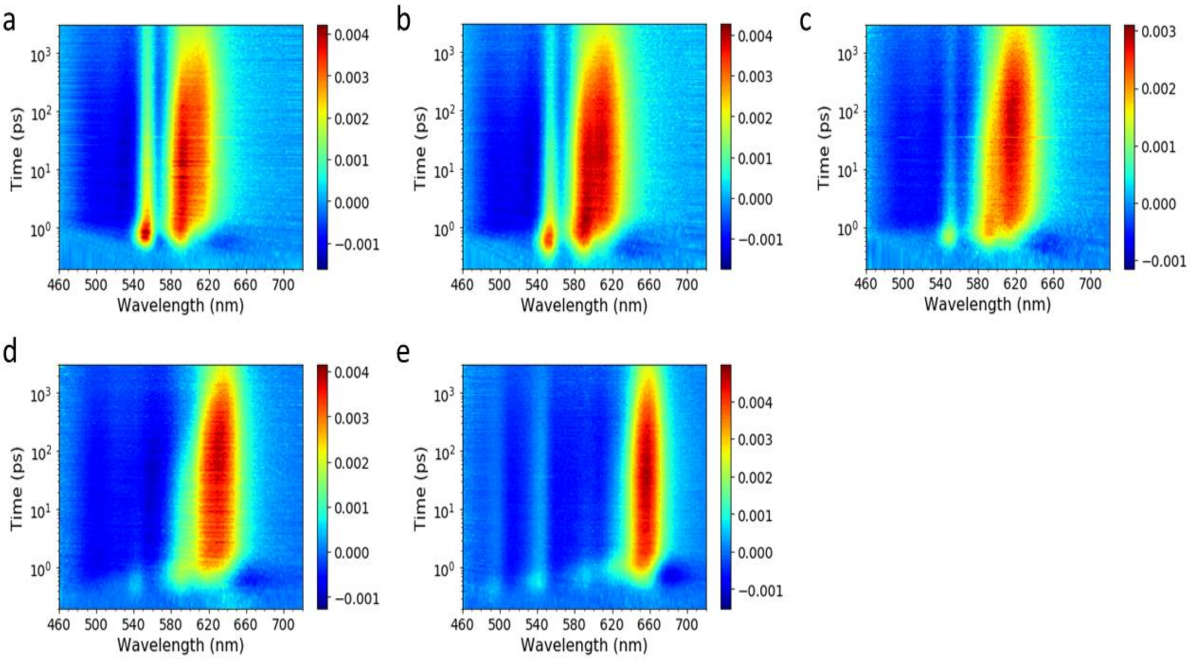

Figure S7. TA spectra (ΔT/T) as a function of probe wavelength and delay time for the $(PBA_xMBZA_{1-x})_2Cs_{n-1}Pb_nI_{3n+1}$ RPP films with x = a) 1, b) 0.75, c) 0.5, d) 0.25, and e) 0.

Determining donor and acceptor QWs by fitting TA kinetics

To establish how the dynamics of funneling is tuned in the alloyed samples, the kinetics of ground-state photobleaching (PB) of different QWs of phase 'n' from TA spectra were fitted with a multi-exponential decay function:

$$Ay = c + \sum_{m}\left[\left(A_m e^{-\frac{x-x_0}{t_m}}\right)\left(1-\text{erf}\left(\frac{-\left(x-x_0-\frac{s^2}{t_m}\right)}{s}\right)\right)\right] \quad \text{(S3)}$$

Here the exponential components are convolved with a gaussian instrument response function. The decays were fitted for up to three decay components with m = 3. t_m and A_m are the lifetime and amplitude (in mOD) of the mth exponential component, respectively. x_0 and c are axis offsets, and s characterizes the risetime due to instrument response. A negative magnitude for A_m indicates signal growth. These sites or acceptors are the emissive sites in the sample. We find that the acceptor QWs have a fast risetime of the order of a few ps after photoexcitation, as shown in Table S3.

The fitted lifetime components are assigned as follows: the slow component t_3, lasting 1000s of ps is assigned to the radiative recombination process in the RPP films, as all n-phases show a decay at this timescale. The funneling of charge carriers to higher n-phases in the films proceeds in two timescales: t_1, which is the fast transfer lasting within a few ps, which is assigned as a population transfer mediated by delocalization of exciton wavefunction leading to coupling between adjacent QWs.[2,4] The slower process t_2 is assigned to charge transfer and has been observed previously at this timescale.[5-7]

Table S3. Results of fitting of TA Kinetics for different QWs 'n'. The Donor and Acceptor were identified by the QWs that show positive and negative values for the amplitude A_1, respectively (Negative A_1 Values in bold).

x	QW 'n'	$t_1(A_1)$	$t_2(A_2)$	$t_3(A_3)$

	(Donor/ Acceptor)			
1	2 (D)	0.6 ± 0.1 ps (1.69 ± 0.07)	36 ± 2 ps (0.47 ± 0.01)	6.9 ± 0.8 ns (0.42 ± 0.01)
	3 (A)	**9 ± 5 ps (-0.94 ± 0.02)**	240 ± 20 ps (0.77 ± 0.03)	5.5 ± 0.7 ns (0.94 ± 0.04)
	4 (A)	**0.5 ± 0.1 ps (-0.91 ± 0.2)**	7 ± 2 ps (-0.24 ± 0.04)	3.1 ± 0.1 ns (1.43 ± 0.01)
0.5	2 (D)	0.5 ± 0.1 ps (0.9 ± 0.2)	7 ± 2 ps (0.22 ± 0.04)	-
	3 (D)	1.0 ± 0.2 ps (0.29 ± 0.06)	150 ± 10 ps (0.42 ± 0.02)	6 ± 1 ns (0.34 ± 0.02)
	4 (A)	0.4 ± 0.1 ps (-1.0 ± 0.2)	9 ± 3 ps (-0.17 ± 0.03)	3.1 ± 0.1 ns (1.28 ± 0.01)
	5(A)	**0.5 ± 0.1 ps (-2.1 ± 0.5)**	14 ± 3 ps (-0.16 ± 0.02)	4.2 ± 0.2 ns (0.90 ± 0.01)
0	2 (D)	0.2 ± 0.1 ps (1.0 ± 0.4)	90 ± 20 ps (0.09 ± 0.01)	-
	3 (D)	0.5 ± 0.1 ps (1.3 ± 0.5)	16 ± 5 ps (0.12 ± 0.02)	2.6 ± 0.3 ns (-0.33 ± 0.01)
	4 (D)	0.4 ± 0.1 ps (2 ± 1)	55 ± 8 ps (0.22 ± 0.01)	2.9 ± 0.4 ns (-0.22 ± 0.01)
	$n \geq 5$ (A)	**11 ± 2 ps (-0.32 ± 0.02)**	580 ± 90 ps (0.9 ± 0.1)	9 ± 3 ns (1.2 ± 0.1)

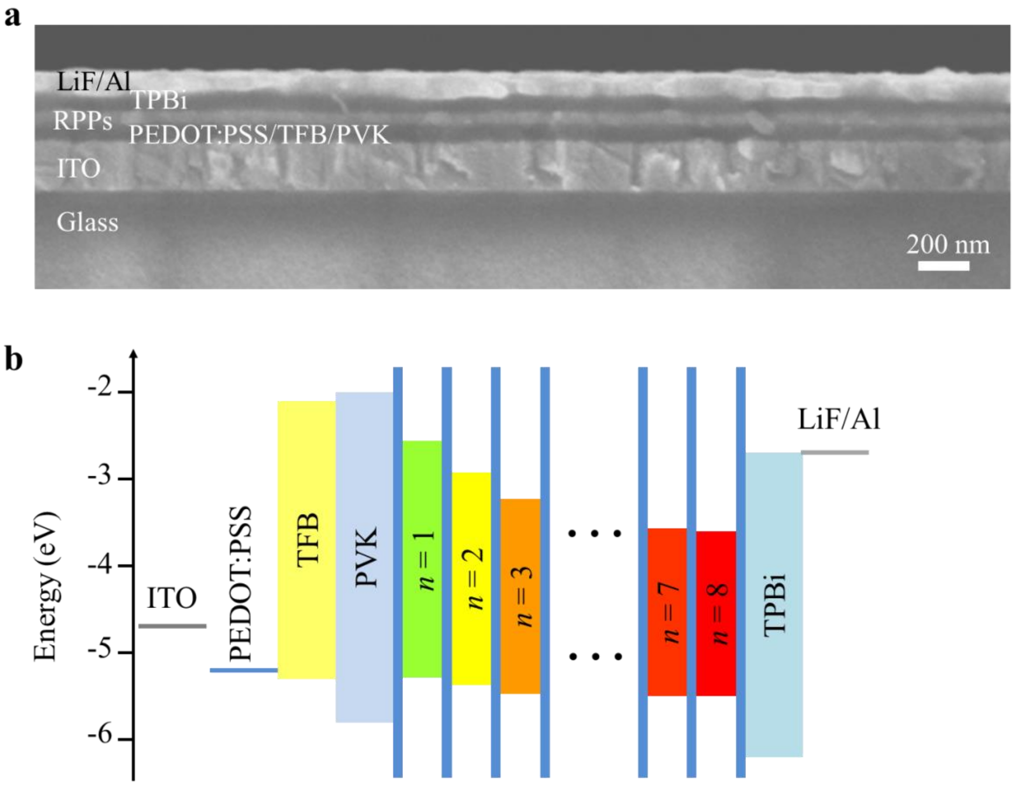

Figure S8. a) Cross-sectional SEM image of a typical PeLED. b) Energy level diagram according to previous reports.[8-11]

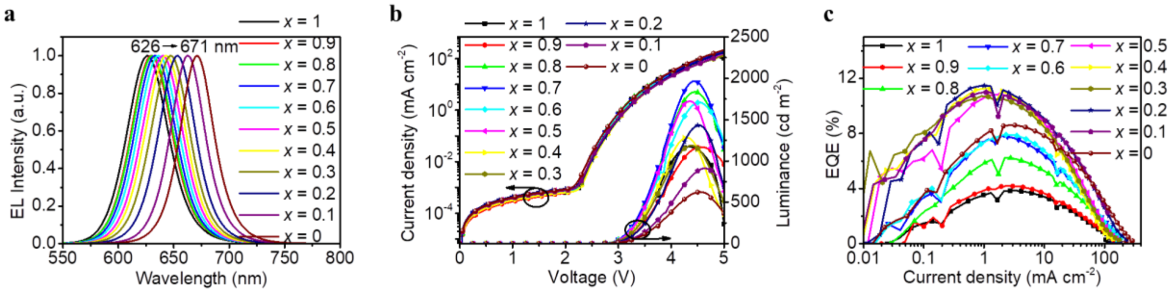

Figure S9. a) Normalized EL spectra, b) current density-voltage-luminance characteristics, and c) EQE values versus current density of the PeLEDs with different alloying ratios of PBA:MBZA.

Table S4. Performance parameters of PeLEDs with different alloying ratios of PBA:MBZA.

x	Peak EQE	Peak current	Maximum	EL peak	FWHM	CIE (*x*, *y*)

	(%)	efficiency (cd A^{-1})	luminance (cd m^{-2})	(nm)	(nm)	
1	3.8	5.2	1173	626	43	(0.67, 0.33)
0.9	4.2	5.0	1169	630	43	(0.67, 0.32)
0.8	6.2	7.2	1835	631	43	(0.68, 0.32)
0.75	7.0	8.0	1927	632	43	(0.68, 0.32)
0.7	7.8	8.4	1966	633	43	(0.68, 0.32)
0.6	7.9	7.7	1709	636	43	(0.69, 0.31)
0.5	10.8	9.4	1724	640	42	(0.69, 0.31)
0.4	11.3	8.7	1284	643	42	(0.70, 0.30)
0.3	10.7	6.9	1187	648	42	(0.70, 0.30)
0.25	11.0	6.3	1206	650	41	(0.70, 0.30)
0.2	11.5	5.7	1431	653	39	(0.71, 0.29)
0.1	11.0	3.4	911	663	37	(0.71, 0.28)
0	8.6	1.7	626	671	36	(0.72, 0.28)

Table S5. Performance summary of recently reported RPP-based red PeLEDs.

Perovskite material	EL peak (nm)	Peak EQE (%)	Maximum luminance (cd m^{-2})	Ref.
$(NMA)_2Cs_{n\text{-}1}Pb_nI_{3n+1}$	689	3.7	440	Adv. Mater. 2017, 1606600
$(BA)_2Cs_{n\text{-}1}Pb_nI_{3n+1}$	638	0.41	390	Adv. Mater. 2018, 1707093
	664	2.81	1231	
	680	6.23	1392	
	690	4.73	186	

$(NMA)_2Cs_{n-1}Pb_nI_{3n+1}$	694	7.3	732	J. Phys. Chem. Lett. 2018, 9, 881− 886
	650	9.5	1000	
	661	11.6	1000	
$(PBA)_2Cs_{n-1}Pb_nI_{3n+1}$	664	13.3	968	ACS Photonics 2019, 6, 587.
	669	11.3	1000	
	680	10.6	100	
**$(PBA_xMBZA_{1-x})_2Cs_{n-1}Pb_nI_{3n+1}$**	**626-671**	**3.8-11.5**	**600-2000**	**This work**

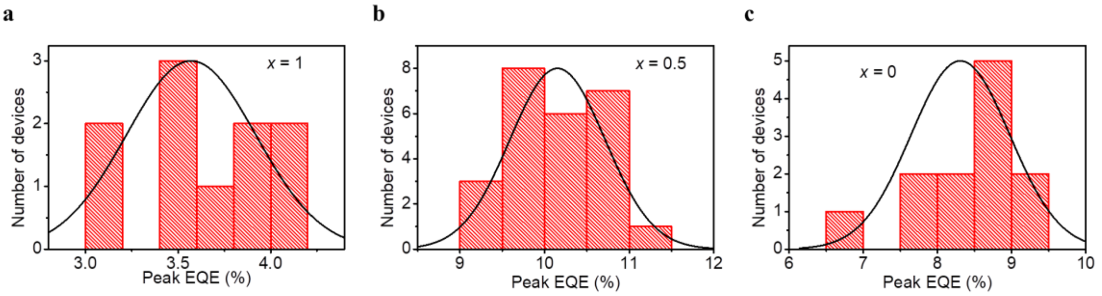

Figure S10. Histograms of peak EQEs for PeLEDs with x = a) 1, b) 0.5, and c) 0.

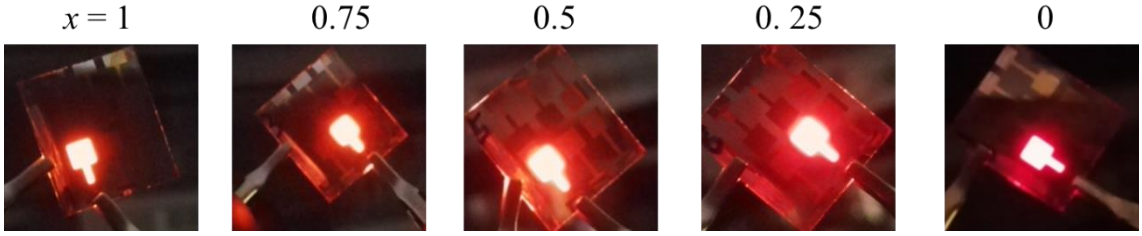

Figure S11. Photographs of working PeLEDs with different alloying ratios of PBA:MBZA at an applied voltage of 4 V.

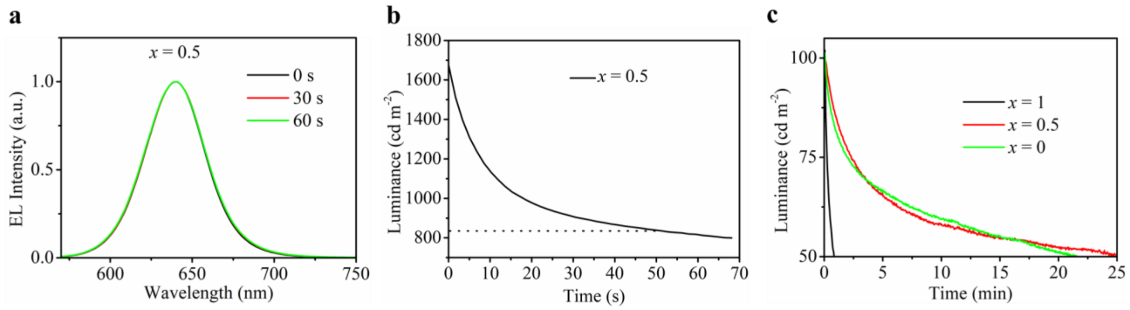

Figure S12. a) EL spectral stability of x = 0.5 device measured at maximum luminance. b) Operational stability of x = 0.5 device measured at maximum luminance. c) Lifetime measurements of PeLEDs with an initial luminance of 100 cd m^{-2}.

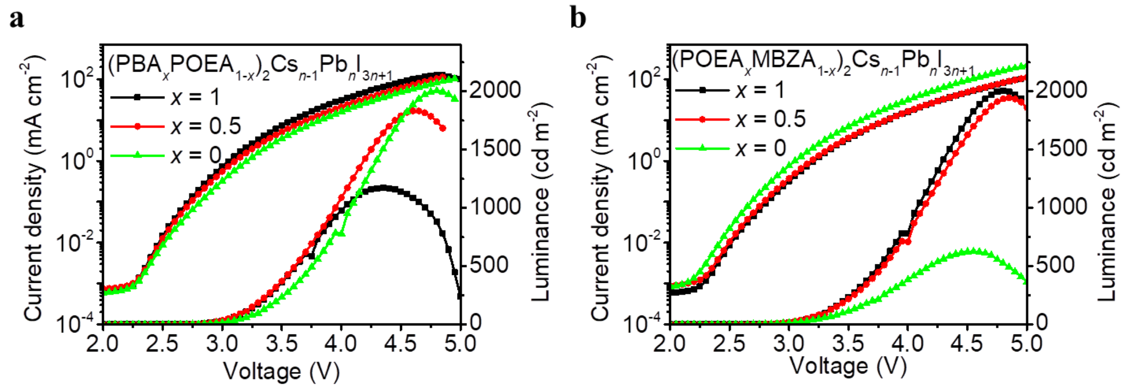

Figure S13. Current density-voltage-luminance characteristics of the PeLEDs based on a) PBA:POEA and b) POEA:MBZA.

Reference

[1] M. Kumagai, T. Takagahara, *Phys. Rev. B* **1989**, *40*, 12359.

[2] D. Giovanni, S. Ramesh, M. Righetto, J. W. Melvin Lim, Q. Zhang, Y. Wang, S. Ye, Q. Xu, N. Mathews, T. C. Sum, *Nano Lett.* **2021**, *21*, 405.

[3] Y. H. Chang, C. H. Park, K. Matsuishi, *J. Korean Phys. Soc.* **2004**, *44*, 889.

[4] G. Xing, B. Wu, X. Wu, M. Li, B. Du, Q. Wei, J. Guo, E. K. L. Yeow, T. C. Sum, W. Huang, *Nat. Commun.* **2017**, *8*, 14558.

[5] J. Liu, J. Leng, K. Wu, J. Zhang, S. Jin, *J. Am. Chem. Soc.* **2017**, *139*, 1432.

[6] A. H. Proppe, R. Quintero-Bermudez, H. Tan, O. Voznyy, S. O. Kelley, E. H. Sargent, *J. Am. Chem. Soc.* **2018**, *140*, 2890.

[7] Q. Shang, Y. Wang, Y. Zhong, Y. Mi, L. Qin, Y. Zhao, X. Qiu, X. Liu, Q. Zhang, *J. Phys. Chem. Lett.* **2017**, *8*, 4431.

[8] Z. He, Y. Liu, Z. Yang, J. Li, J. Cui, D. Chen, Z. Fang, H. He, Z. Ye, H. Zhu, N. Wang, J. Wang, Y. Jin, *ACS Photonics* **2019**, *6*, 587.

[9] M. Yuan, L. N. Quan, R. Comin, G. Walters, R. Sabatini, O. Voznyy, S. Hoogland, Y. Zhao, E. M. Beauregard, P. Kanjanaboos, Z. Lu, D. H. Kim, E. H. Sargent, *Nat. Nanotechnol.* **2016**, *11*, 872.

[10] C. Sun, Y. Jiang, M. Cui, L. Qiao, J. Wei, Y. Huang, L. Zhang, T. He, S. Li, H.-Y. Hsu, C. Qin, R. Long, M. Yuan, *Nat. Commun.* **2021**, *12*, 2207.

[11] N. Wang, L. Cheng, R. Ge, S. Zhang, Y. Miao, W. Zou, C. Yi, Y. Sun, Y. Cao, R. Yang, Y. Wei, Q. Guo, Y. Ke, M. Yu, Y. Jin, Y. Liu, Q. Ding, D. Di, L. Yang, G. Xing, H. Tian, C. Jin, F. Gao, R. H. Friend, J. Wang, W. Huang, *Nat. Photon.* **2016**, *10*, 699.
